# Cognitive Trajectories Following Acute Infection in Older Patients With and Without Cognitive Impairment: An 1-Year Follow-Up Study

**DOI:** 10.3389/fpsyt.2021.754489

**Published:** 2021-12-16

**Authors:** Ana Rita Silva, Patrícia Regueira, Ana Luísa Cardoso, Inês Baldeiras, Isabel Santana, Joaquim Cerejeira

**Affiliations:** ^1^Centro de Neurociências e Biologia Celular, University of Coimbra, Coimbra, Portugal; ^2^Serviço de Psiquiatria, Centro Hospitalar e Universitário de Coimbra, Coimbra, Portugal; ^3^Faculdade de Medicina, Universidade de Coimbra, Coimbra, Portugal; ^4^Serviço de Neurologia, Centro Hospitalar e Universitário de Coimbra, Coimbra, Portugal

**Keywords:** acute infection, cognitive impairment, delirium, dementia, cognitive trajectories

## Abstract

**Introduction:** Dementia is a known risk factor for both delirium and acute systemic infections which may also play a significant role in promoting or accelerating neurodegenerative disease. Infections are both the main causes of hospitalization of dementia patients and can be a major precipitant of delirium but currently it is not possible to predict the risk of cognitive decline in older patients exposed to acute infection.

**Objectives:** We aimed to determine the level of cognitive change at 1-year follow up in individuals with different patterns of cognitive function (dementia, delirium, delirium superimposed on dementia) at the time of their hospitalization due to a systemic infection and to correlate these cognitive patterns with clinical status variables.

**Methods:** We recruited 53 hospitalized geriatric patients with a systemic infection, and we collected 12-months follow up data for 34 patients. These patients were classified in four groups: no cognitive impairment (controls—C), delirium only (D), dementia only (Dem), and delirium superimposed to dementia (DD). Cognitive performance was measured by change in score on the Montreal Cognitive Assessment (MoCA) and delirium was identified using Confusion Assessment Measure (CAM). We examined performance on the MoCA in the first year after hospitalization, controlling for demographic characteristics, coexisting medical conditions, and type of infection.

**Results:** For the 34 patients to whom follow-up data was available, delirium presence in individuals with prior dementia (DD group) was associated with a negative mean change score of 3-point (*p* < 0.02) at 1 year follow up, whereas dementia patients without delirium had a mean change score of 1.5-point lower at 12-months (*p* = 0.04), when comparing follow-up and baseline MoCA scores. Cognitively healthy patients did not significantly decrease their MoCA score at follow-up (*p* = 0.15). MoCA and NPI scores during hospitalization were significantly correlated with the level of cognitive decline in the four groups (*r* = 0.658, *p* < 0.01 and *r* = 0.439, *p* = 0.02, respectively).

**Conclusions:** Premorbid dementia and delirium superimposed on dementia during hospitalization in older patients with acute infections predict cognitive decline at 1 year following admission. Taken together, our findings suggest a pathophysiological interaction between neurodegenerative changes, acute infection, and delirium.

## Introduction

Dementia refers to a number of disorders affecting the brain, including Alzheimer's disease and other neurodegenerative conditions, which manifest by a progressive and global decline of cognitive function, altered behavior and ability to perform daily activities (1). Pneumonia and urinary tract infections are the main cause of hospitalization of dementia patients (2) and infections are a major precipitant of delirium in this population (3). Dementia and delirium are particularly associated with increased likelihood of hospital acquired complications (4, 5), longer hospital stay (6) as well as higher risk of functional decline, hospital readmission and death following discharge (7, 8). Delirium is more common in individuals with prior dementia and has a direct impact on cognitive function increasing the risk of further cognitive decline (9–11).

Acute systemic infections may play a significant role in promoting/accelerating neurodegenerative disease although the mechanisms for these associations are poorly understood (12). Data derived from animal studies show that acute systemic inflammation elicits a prompt neuroinflammatory reaction in the CNS largely mediated by activated microglial cells (13). In animal models of chronic neurodegeneration, a peripheral immune challenge has been shown to induce irreversible cell loss and progression of neurodegenerative disease (14–16). Evidence exploring the contribution of acute systemic inflammation (e.g., infection) in the cognitive trajectory of older people remains very limited. Baseline serum levels of inflammatory markers have been associated with a risk of cognitive decline (17). Acute systemic inflammatory events have been associated with a 2-fold increase in the rate of cognitive decline over a 6-month period (18) and patients with AD who experienced an infection and/or delirium had accelerated cognitive decline (19). Acute infections are amongst the leading causes of hospitalization that act as triggers of delirium onset, despite few studies having been published reporting infection hospitalization-related delirium and deepening the understanding of long-term effects on cognition of this kind of delirium inductor (20). Some studies suggest that viral infections, even if not severe enough to warrant hospitalization and in the absence of a delirium episode, contribute to long term cognitive decline (21–23).

Taken together, this evidence suggests that acute systemic inflammation can have a major impact in the brain and can mediate the relation between increasing age, neurodegeneration, and delirium as well as the relation between delirium and cognitive deterioration at long term. Yet, there is a lack of longitudinal studies on cognitive outcomes of older people following an acute infection, that correlate long term patterns of cognitive decline with clinical variables and infection-related data, that could offer a deeper understanding on the relationship between these events. The understanding of the effects of an acute systemic infection in the cognitive trajectory in patients with dementia and non-dementia patients is particularly relevant in the current pandemics, with the SARS-COV-2 virus already demonstrating to be associated with more episodes of delirium in hospitalized and ventilated patients (24). Consequently, in this study, we aimed to examine the change in cognitive function in older adults, 1 year after an acute infection requiring hospitalization. Specifically, our primary goal was to determine if cognitive impairment prior to hospitalization due to an infection (i.e., dementia) and/or the emergence of delirium during hospitalization significantly increased the risk of subsequent cognitive deterioration at 12-months. As secondary outcomes, we also examined cognitive change at 1-year and subdomains of cognitive change at both time points, and we examined the role of clinical and infection-related variables as predictors of cognitive change.

## Materials and Methods

### Participants

All individuals aged over 65 years old with unplanned acute admission to Internal Medicine wards between January 2019 and February 2020 with an acute systemic infection were eligible for inclusion in the study. Acute systemic infection was defined as an infection not involving directly the central nervous system with C-reactive protein plasma levels superior to 1 mcg/mL and requiring treatment with antibiotics. Exclusion criteria included patients admitted for <48 h and those not able to undergo neuropsychological assessment. Written informed consent was obtained for each patient and/or their legal representatives according to the ethical procedures submitted and approved by the Ethical Committee of Centro Hospitalar Universitário de Coimbra (Ethics approval Ref. CHUC-065-18).

### Procedures

#### Clinical Assessment

##### Diagnosis of Dementia and Prevalent Delirium During Hospitalization—Baseline Assessment

All participants were assessed within 72 h of admission by a psychiatrist and were first screened with the Richmond Agitation and Sedation Scale (RASS) (25) to assess the level of consciousness. Participants with RASS > −3 were assessed with the Confusion Assessment Method (CAM) (26) during a formal cognitive test with the validated Portuguese version of Montreal Cognitive Assessment (MoCA) (27). Participants were reassessed prior to discharge (<12 h before discharge) with MoCA test, being all free from delirium, to establish the level of cognitive performance to be used in the study analysis. Information about the premorbid cognitive function was derived from the IQCODE, a validated screening tool about the daily cognitive performance. Based on a 5-point scale from “much improved” to “much worse,” the Portuguese version of the IQCODE (28) has a cutoff-score of 3.60 (sensitivity 60%, specifity 78%) to point for the presence of previous dementia, and we used this threshold for the comparison of the cognitive performance with the control group. IQCODE was presented to relatives familiar with the patient and supported also by a review of clinical records. Dementia-related information collected included family history of dementia and duration of dementia symptoms before diagnosis. Participants were classified in four groups: without cognitive impairment (controls—C), dementia (dementia only—Dem), without cognitive impairment but with delirium onset during hospitalization (Delirium only—D) and delirium superimposed on dementia (DD). Data from laboratorial results and clinical records were collected about: demographic variables (age, gender, education, place of residence); current medication list, smoking habits, alcohol consumption and previous psychiatric or neurologic diseases; severity of chronic comorbidities (Charlson Comorbidity scale) (29) type of acute medical illness classified according to ICD-10 (30); functionality status measured with Barthel Index (BI) (31); clinical status severity (using the Sequential Organ Failure Assessment (SOFA) (32) length of hospital stay and mortality at 12 months; number and severity of neuropsychiatric symptoms prior to hospital admission, using the Neuropsychiatric Inventory (NPI) (32). Patients included in the study were daily assessed for the development of new episodes of delirium, based on all sources of information available, using RASS and CAM (33). The classification of patients with delirium was made if they developed at least one episode of delirium (irrespective of its severity) during hospitalization.

##### Assessment at 12 Months Following Hospitalization

Participants were reassessed after 12 months with the MoCA, and by both checking clinical records from the previous 12 months and interviewing the available caregiver, we confirmed the absence of other hospitalizations and absence of other infection requiring medical assistance during the previous 12-months.

The primary outcome of this study was the rate of cognitive change, as measured by changes in MoCA scores over the 12-months. The MoCA is an extremely well-validated and widely used brief cognitive measure sensitive to cognitive change due to neurodegeneration and briefly measuring domains of Visuospatial/Executive, Naming, Memory, Attention, Language, Abstraction, Delayed Recall and Orientation. MoCA scores range from 0 to 30, with lower scores indicating cognitive impairment.

### Statistical Analyses

The study cohort was described by mean (standard deviation) for continuous characteristics, number (percent) for categorical and by median [interquartile range] for characteristics with skewed distribution. To conduct the uni and multivariate analyses of variance (ANOVAs, MANOVAs and ANCOVAs), Skewness (Sk) and Kurtosis (Ku) were analyzed (|Sk| < 3; |Ku| < 8), revealing a non-violation of the normality assumption (34). Linear mixed models were applied for two purposes: First, we looked for potentially confounding covariates at SV1. We started with a priori chosen variables based on clinical judgement and previous research. These were: Premorbid cognitive impairment (determined by the IQCODE), Charlson Comorbidity Index (CCI), hospital length of stay, severity of delirium symptoms (DRS-98-R). The cognitive outcome variables MoCA at follow-up and cognitive change (Difference between MoCA follow-up and MoCA at baseline) were chosen as dependent variables. We estimated effects including confidence intervals. In addition to univariate analysis, a multivariable analysis was performed, which adjusted for characteristics known to be associated with cognitive decline including age, education, sex, infection severity, functional status, and premorbid cognition. In all instances, findings were summarized using the odds ratio (OR) and corresponding 95% confidence interval (CI). All reported *P*-values were two sided, and general significance level was set at α = 5% g. Bonferroni-Holm correction for multiple comparisons was used as appropriate. The analyses were performed using IBM SPSS version 24 (SPSS Inc., Chicago, IL, USA).

## Results

### Sample Characteristics

**Figure 1:**
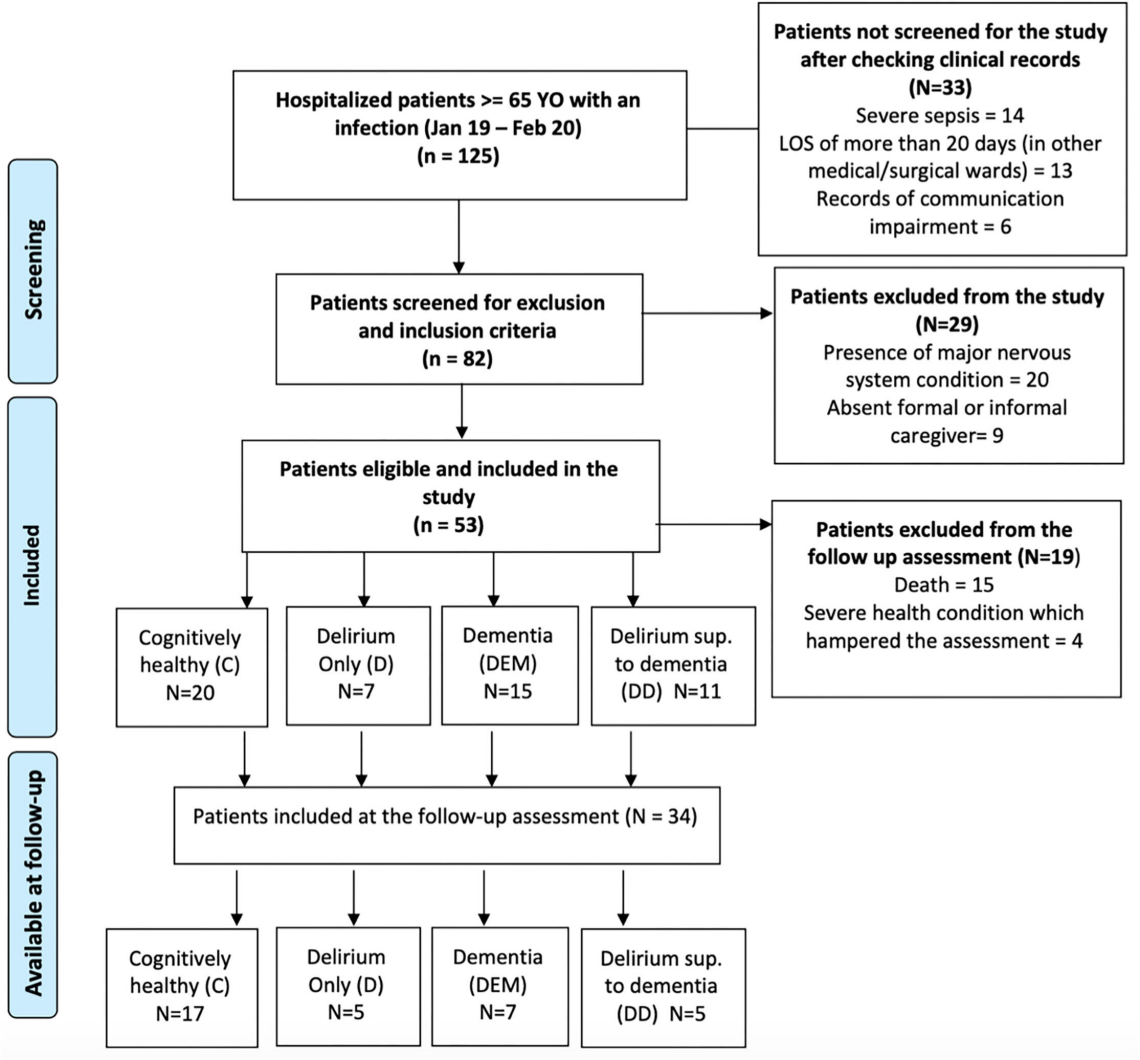
Enrolment flowchart of the patients during hospitalization and at follow-up.

From an initial sample of 82 patients eligible for inclusion in the study, 20 were excluded due to the presence of a major nervous system condition and 9 were excluded due to the lack of a formal or informal caregiver who could provide information regarding premorbid functional and cognitive status (Figure 1). Therefore, 53 patients (Mean age 83.6, SD = 7.12, age range 66–96) underwent full baseline assessment during hospitalization but only 34 patients were fully assessed both at baseline and at 12-months follow-up. Throughout the observation period, 15 patients died, the majority from the cognitive impaired groups [i.e., 6 from the DEM group (42.9%), 3 from the D group (40%) and 5 from the DSD group (45.5%)] with only one patient dead in the C group. Four patients refused to be reassessed and 34 patients were reassessed at follow-up (total dropout rates per group was—Controls −15%; Dementia = 50%; Delirium = 37.5%; Delirium superimposed to dementia = 55%). We found significant differences in the mortality rates across groups, with DEM, DSD, and D groups presenting higher mortality rates than the C group (*p* = 0.025). Despite the high mortality rates, the final sample demographic (age, education, gender) and clinical characteristics (type of infection, infection severity, length of stay) did not differ statistically from the initial sample.

The mean age of the final sample was 81.4 years (SD = 7.18, age range 66–95), mostly females (62%) hospitalized due to respiratory tract infections (71%). We included 7 participants with prior dementia (DEM), 5 with delirium during hospitalization without prior cognitive impairment (D), 5 with delirium superimposed on dementia (DSD) and 17 who were cognitively healthy (CH). No differences were found for the demographic variables, type of infection, infection severity through C-reactive protein and SOFA score, and comorbidity index between these groups. Most of the sample were taking at least one drug active on CNS, without between groups differences, and no differences in MoCA scores (MoCA 1 and MoCA 2) were found when grouping the sample according to this variable [MoCA 1—*t*_(32)_ = −0.1.61, *p* = 0.87; MoCA 2—*t*_(32_) = −0.087, *p* = 0.931]. DEM and DSD groups had a similar cognitive performance which was significantly worse than the CH group (Table 1). Pre-admission functional status (measured with Barthel index) was significantly lower in DEM (*p* = 0.02) and DSD (<0.01) groups compared to the CH and D groups.

**Table 1:** Baseline clinical features of the participants who completed the study (*N* = 34).

	**Cognitively healthy (C)** ***N*** **= 17**	**Dementia (DEM)** ***N*** **= 7**	**Delirium (D)** ***N*** **= 5**	**Delirium superimposed on dementia (DD)** ***N*** **= 5**	**Sig**.
Age	80.72 (1.91)	80.86 (2.65)	81.00 (2.67)	85.75 (2.17)	0.53
Gender (female) *N* (%)	10 (50.0)	10 (66.7)	5 (71.4)	7 (63.6)	0.56
Years of formal education *N* (%)	10 or more = 3 (15%) 5–9 = 2 (10%) 1–4 = 13 (65%) <1 = 2 (10%)	1–4 = 6 (40%) <1 = 9 (60%)	1–4 = 5 (71.4%) <1–2 (28.6%)	1–4 = 5(45.5) < 1 = 5 (45.5)	0.07
Residence	Own housing 19 (95%) Nursing home 1 (5%)	Own housing 10 (66.7%) Nursing home 5 (33.3%)	Own housing 6 (85.7%) Nursing home 1 (14.3%)	Own housing 9 (81.8%) Nursing home 2 (18.2$)	0.21
Barthel Index (BI)	82.9 (19.2)	47.5 (31.5)^*^	77.0 (29.7)^*^	33.6^*^ (30.8)	**<0.01**
SOFA score (Mean/SD)	2.61 (1.58)	2.71 (0.48)	2.00 (1.23)	3.75 (1.90)	0.34
Comorbidity Index (CCI Mean/SD)	5.56 (1.72)	5.15 (1.87)	4.80 (1.32)	7.72 (2.07)	**0.08**
MoCA (Mean/SD)	18.2 (0.9)	10.71^*^ (1.4)	7.0 (1.6)^*^	7.5^*^ (1.8)	**<0.01**
IQCODE (Mean /SD)	1.56 (1.2)	4.12 (1.3)^*^	2.01 (1.98)	4.36 (1.9)^*^	**0.021**
Neuropsych symptoms (NPI, Median/IQR)	12.00 (20)	67.50 (30)^*^	24.00 (21)	84.50 (27)^*^	**<0.01**
DRS-98-R (Delirium Severity, Mean/SD)	–	–	15.83 (1.48)	16.20 (8.93)	0.90
Delirium length (Mean n° of days with delirium/SD)	–	–	2.56 (0.67)	3.01 (1.99)	0.78
Type of infection	Respiratory Tract 14 (70.0) Urinary Tract 4 (20.0) Bacteremia 1 (5.0) Pericardial tract 1. (5.0)	Skin and soft tissues 2 (13.3) Respiratory Tract 12 (80.0) Urinary Tract 1 (6.7)	Skin and soft tissues 1 (14.3) Respiratory Tract 6 (85.7)	Respiratory Tract 6. (54.6) Urinary Tract 5 (45.5)	0.11
C-reactive protein levels	5.96 (6.61)	2.74 (9.9)	3.48 (3.32)	4.75 (3.20)	0.24
CNS drugs usage (Yes/No/%)	Yes = 14 (82%) No = 3 (18%)	Yes = 7 (100%)	Yes = 4 (80%) No = 1 (20%)	Yes = 5 (100%)	0.37
Length of stay (Median/IQR)	7 (3)	7.5 (7)	9.0 (8)	7 (6)	0.62
Pain score	4 (5)	2 (2.5)	2 (3.5)	2.5 (4.5)	0.46

* *Sig. <0.01.*

*Categorical variables were analyzed using Chi-Square statistics (Gender, Formal Education, Residence, Type of Infection, CNS drugs usage) and remaining variables groups were compared using ANOVA statistics.*

*Bold values are statistically significant differences between groups sig. p < 0.05*.

**Figure 2:**
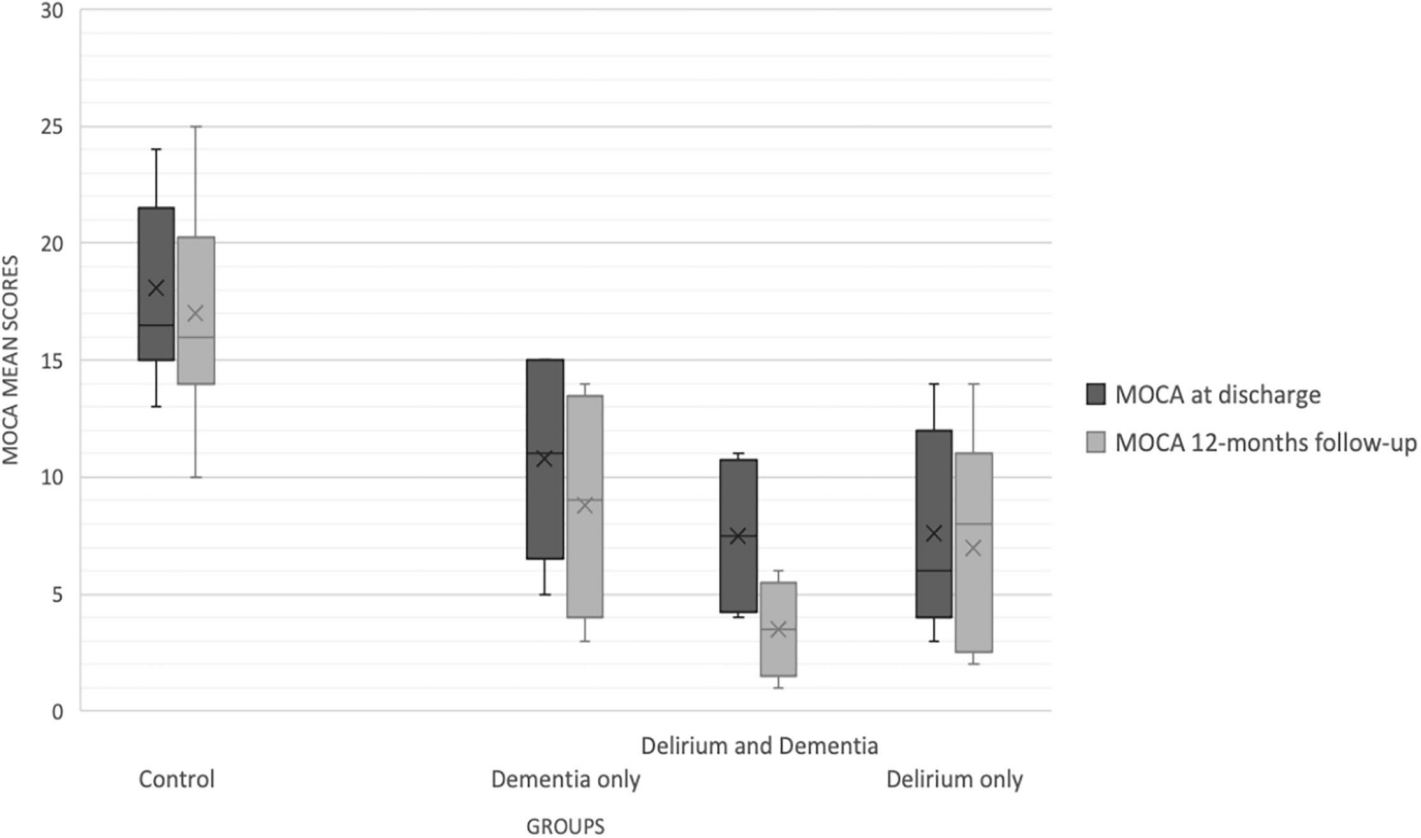
MoCA scores during hospitalization and at 12-months follow-up.

Differences in cognitive performance between groups persisted at follow-up, with *Post-Hoc* analyses finding lower MoCA scores both in DEM, DSD and D relative to Controls (*p* = 0.003, *p* < 0.001 and *p* = 0.023, respectively) (Figure 2).

### Cognitive Change at 12-Months Following Hospitalization Due to an Acute Systemic Infection

**Figure 3:**
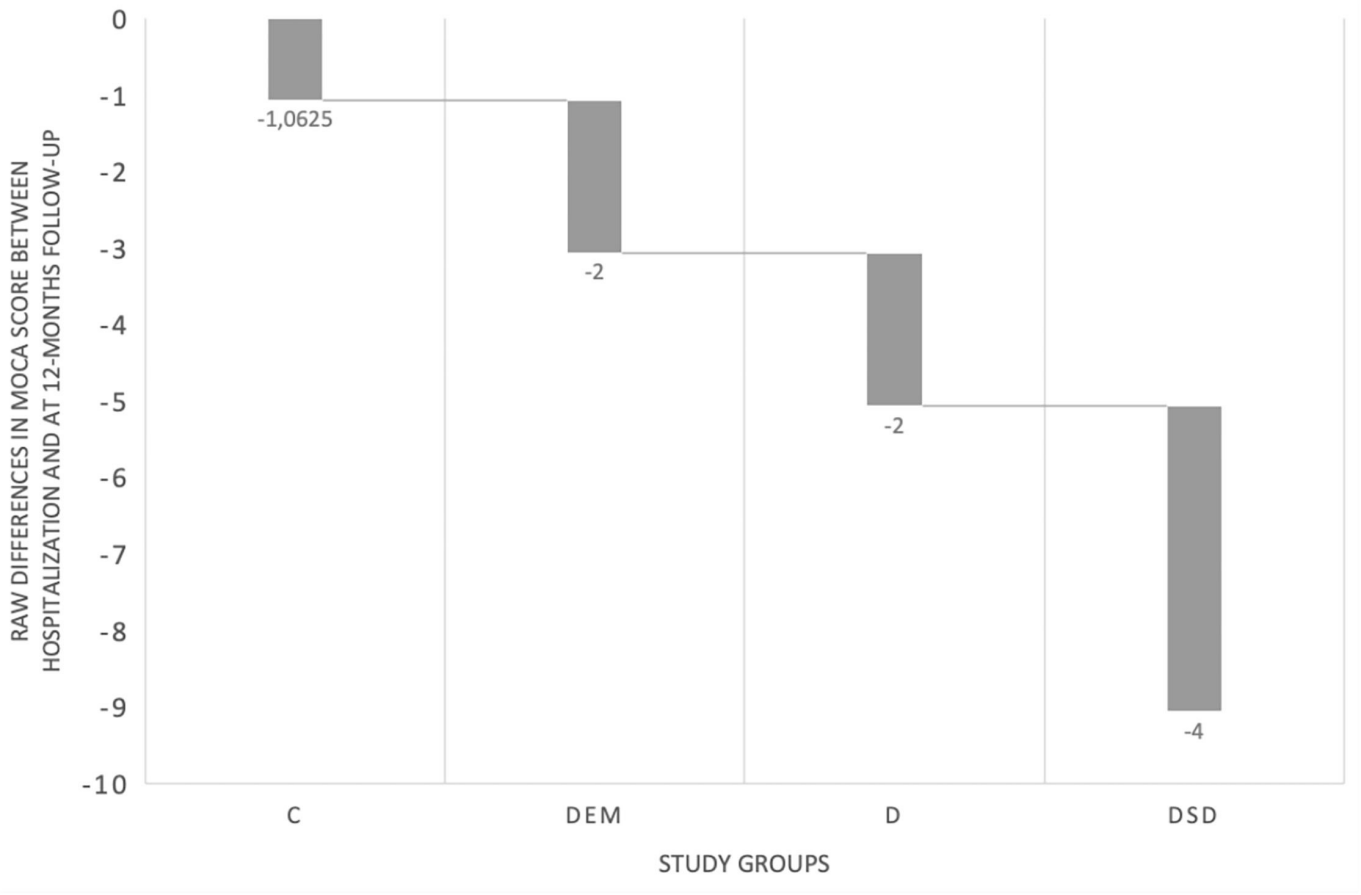
MoCA scores mean change in the four study groups.

As depicted in Figure 2, MoCA scores decreased at follow-up in the global sample (*F* = 4.213, *p* = 0.01), but a significant cognitive change (differences between MoCA 1 and MoCA 2) was observed only in participants with DSD (−4.01 points, CI 95% −1.98 and −6.89, *p* = 0.001) and in those with Dementia (−1.85, CI 95% −2.49, −1.22, *p* = 0.02). After adjusting for a set of relevant variables that could influence cognitive performance at follow-up (age, type of infection, length of stay, comorbidity index, IQCODE score, premorbid NPI and Barthel Index; performing an ANCOVA), and adjusting for multiple comparisons (Bonferroni), we identified a significant decrease in the cognitive status only for the DSD (−6.18 points, CI 95% −10.68 to −1.45, *p* < 0.003). Mean MoCA score change is depicted in Figure 3.

We examined the raw scores in MoCA subdomains (executive, attention, language, memory and orientation) both at baseline (during hospitalization) and at follow-up (Table 2). Secondly, we examined the differences across the groups in the two assessments, and no differences were found in the DEM, D and DSD groups. We noticed differences in the C group for the performance in attention, which improved 12-months following hospitalization [*t*_(16)_ = −2.784, *p* = 0.014].

**Table 2:** Performance in MoCA subdomains, at baseline and following 12-months.

**MOCA domains**	**Cognitively healthy (C)**		**Dementia (DEM)**		**Delirium (D)**		**Delirium Superimposed to** **dementia (DSD)**	
	**Baseline**	**12-months**	**Baseline**	**12-months**	**Baseline**	**12-months**	**Baseline**	**12-months**
Executive	2.94 (2.01)	3.19 (1.75)	1.09 (0.83)	1.28 (0.65)	1.00 (0.3)	1.33 (0.58)	0.5 (0.2)	0.2 (0.45)
Attention	2.24 (1.12)	5.07^*^ (1.43)	1.82 (1.48)	2.00 (1.34)	1.67 (1.53)	3.00 (0.03)	0.4 (0.54)	0.4 (0.54)
Memory	1.13 (1.5)	1.9 (1.7)	0.18 (0.4)	0.81 (1.4)	0.7 (1.2)	1.67 (1.5)	0.6 (1.34)	0.1 (0.4)
Language	4.3 (1.4)	4.2 (1.05)	2.17 (1.52)	2.08 (1.68)	1.8 (1.8)	2.2 (1.5)	2.00 (1.22)	2.2 (1.09)
Orientation	5.8 (0.54)	5.4 (1.2)	4.00 (1.89)	3.18 (1.84)	2.67 (3.05)	2.33 (0.58)	1.8 (1.3)	1.4 (1.1)
Total	19.3 (3.4)	18.27 (3.65)	9.08 (3.6)	8.1 (3.93)	7.66 (3.93)	8.00 (4.9)	5.6 (3.06)	3.5^*^ (2.08)

* *Paired samples t-test statistics sig. >0.01 after correction for multiple comparisons*.

### Strength of the Relationship of Delirium and Other Clinical Variables in the Cognitive Change at 12-Months

After examining differences between groups, we explored the relation of relevant clinical features with the cognitive performance of these groups after 12-months of hospitalization with an infection. The correlation matrix held significant and positive correlations between of the level of functionality (*r* = 0.609, *p* = 0.003), premorbid cognition (IQCODE *r* = 0.714, *p* = 0.001) and MoCA scores during hospitalization (*r* = 0.960, *p* < 0.01) and the cognitive status at follow-up (MoCA 2). Additionally, the presence of delirium (rho = −0.647, *p* < 0.01) during hospitalization as well as the presence of dementia (rho = −0.594, *p* < 0.01) correlated significantly and negatively with MoCA 2. The type of infection and the remaining clinical variables did not correlate with cognitive status at follow-up. The cognitive change (difference between MoCA 2 and MoCA 1), correlated negatively with MoCA scores at discharge and the cognitive change score (*r* = −0.587, *p* = 0.002) and positive and strong associations between delirium onset during hospitalization (rho = 0.472, *p* = 0.02), premorbid cognitive impairment (IQCODE *r* = 0.574, *p* = 0.002) and neuropsychiatric symptoms—NPI (*r* = 0.526; *p* = 0.006).

To examine the impact of delirium onset during hospitalization due to an infection as a predictor of cognitive decline at 1-year follow up, we performed odds-ratio analyses between delirium (present or absent) and decline in 3-points on MoCA at follow-up (Cognitive change 3 points—yes or no). According to our data, 55.6% of the patients developing delirium during hospitalization presented at least 3-points less in cognitive performance after 1 year, and individuals developing delirium had 9.1 more chances of having this significant cognitive deterioration than individuals who did not develop delirium (OR 9.17, CI 95% 1.54–54.59). Similar odds ratios were found when considering only a 2-point decline in MoCA (OR 11.00, CI 95% 1.39–111.32).

## Discussion

Acute systemic inflammation can have a major impact in the brain and can mediate the relation between increasing age, neurodegeneration and delirium as well as the relation between delirium and cognitive deterioration at long term. In a typical acute medical ward, there may be dozens of patients infected with bacteria and other pathogens, but the magnitude of the innate immune responses will vary considerably, spanning from having no observable inflammation to the release of proinflammatory cytokines that mediate tissue injury and delirium. Thus, currently it is not possible to predict the risk of cognitive decline in subjects with dementia exposed to acute infection and there is a scarcity of data regarding the impact of acute systemic infections in the cognitive function of older patients. We, therefore, evaluated a homogeneous sample of patients hospitalized with an acute systemic infection who were classified in 4 groups (according to their cognitive status) with similar levels of C-reactive protein, disease severity and comorbidity burden. After observing their cognitive trajectories during 12-months we found 3 distinct profiles: (a) patients with normal cognition during hospitalization who remained cognitively stable at 12-months; (b) patients presenting with cognitive dysfunction (delirium or dementia) during hospitalization who remained cognitively stable during 12-months; and (c) patients presenting with delirium superimposed on dementia during hospitalization who deteriorated their cognitive function at 12-months.

Differentiation between dementia and delirium in acutely-ill medical patients is often difficult due to a substantial clinical overlap between both conditions (35). Accordingly, we found that participants with prior dementia, those with delirium (without dementia) and with delirium superimposed on dementia had similar cognitive performance when discharged from the hospital. Moreover, older patients with normal cognitive function and those with dementia prior to hospitalization (but no delirium) presented with deficits in attention (as assessed with MoCA) during hospital stay. Therefore, even with specific attention tests, such as the Months of the Year Backwards Test, differentiation between dementia and delirium can be challenging (36) supporting the concept of “cognitive spectrum disorders” as a generic term for older patients admitted to hospital presenting with cognitive impairment irrespective of the specific diagnosis (37).

Older people admitted to hospital with an acute infection and presenting with a normal cognitive function remained stable for 12 months. Similarly, patients with dementia prior to hospital admission who did not develop delirium during hospitalization had a stable cognitive trajectory during 12-months with persisting deficits in attention and orientation. In contrast to the idea that delirium has a benign course, participants without prior dementia who developed delirium during hospital stay had persistent cognitive impairment in 1 year following hospitalization without recovering the premorbid cognitive function. The persistence of delirium for 2–4 months, known as “persistent delirium,” has been described following hospital discharge (38) although in our study the time period is substantially greater and can represent an irreversible sequela following delirium. Moreover, incident delirium in older patients with prior dementia hospitalized with acute infections was associated with a significant cognitive decline at 12-months. Delirium occurrence during hospitalization was associated with a 4-fold higher likelihood of cognitive decline at 12-months compared to those not experiencing delirium. The stability of cognitive change in individuals with prior dementia who did not developed delirium is in line with other studies suggesting a 1-year cognitive change of maximum 2-points in a cognitive screening test (MMSE) (39, 40) (MoCA) (41), partially due to the lack of sensitivity of these measures to significant cognitive change in <2 years follow-up.

From a clinical perspective, these findings support the hypothesis that an acute systemic infection can have an impact on cognitive function both in the short and long term. However, according to our study, this effect at long term was particularly significant in patients with ongoing dementia who developed delirium symptoms during hospitalization. An important body of evidence shows that sepsis is associated not only with acute cognitive dysfunction but also with permanent damage in the CNS, particularly in regions commonly affected by neurodegeneration such as the hippocampus and frontal cortex (42). A recent meta-analysis which included 23 studies confirmed an association between delirium and persistent cognitive impairment at 3 months or longer after delirium onset (10). This association was found both in surgical and non-surgical settings although the heterogeneity between studies was high and there was a lack of sufficient number of patients with previous dementia. Moreover, only three studies in non-surgical settings were included. Recently, a study with nursing home residents who were hospitalized due to an infection reported immediate and persistent cognitive decline for up to at least six quarters after hospitalization, particularly in dementia patients and in those with more than 85 years (43). The cognitive pathways of our delirium only group also suggest that, despite these patients had normal cognitive performance before the infection-related hospitalization (according to IQCODE), their cognitive status was persistently lower both before discharge (after delirium symptoms were resolved or only under subclinical delirium symptoms) and at 12-months follow up. However, the interpretation of these pathways is challenging and demand a detailed assessment of potential factors mediating the impact of infection and hospital-related factors in this group of patients. The small sample size of this group prevents any further interpretations and thus future studies with adequate sample sizes in the four groups are needed to elucidate this issue.

Our findings agree with substantial data compiled in the referred meta-analysis (10), suggesting that delirium onset in critical illnesses increases the progression of dementia clinical status. However, our data deepens the understanding of this association between delirium and dementia worse outcomes in a sample of older adults with the same level of acute systemic infection pathway, thus enabling us to clarify this association excluding a wide set disease-related sources of bias. Additionally, the inclusion of a control group without premorbid cognitive impairment, where we identified single effects of hospitalization (e.g., attention impairment) offers us an opportunity to distinguish these effects from those associated with cognitive impairment variables, constituting another relevant asset of this study. Some literature regarding hospitalization effects in older adults' cognition have already described attention and recoverable global cognitive dysfunction (44, 45), but it is important to highlight that the current study did not used a specific measure of attention, and our findings are based in MoCA subdomains, which prevents further considerations concerning this cognitively healthy group.

The mortality rates identified in our study are in line with the previous findings associating cognitive spectrum disorders during hospitalization with higher rates of mortality at the medium term (46, 47). Both DEM, DSD and D groups presented with significantly higher mortality rates at 12-months compared with the cognitively healthy group (C) and this largely explained the highest values dropout rates at 12-months within these groups (50 and 55%, respectively, corresponding to mortality rates and inability to be reassessed due to communication/functional problems). Not surprisingly, other researchers reported that pre-existing dementia and delirium superimposed on dementia strongly predict worse outcomes at 12 and 24 months (37, 47, 48), with little variation between these different cognitive spectrum disorders.

According to the stress-diathesis model patients with dementia are predisposed to the development of delirium because of acute precipitants (related to medical illness, pharmacotherapy, and contextual factors) in a vulnerable brain (49–52). In the specific case of acute systemic infections, we have proposed that a deregulated neuroinflammatory response with exaggerated microglial activation to acute systemic inflammation not only underlie the transient symptoms of delirium but also induce long lasting structural damage with neuronal cell loss (13). Similarly, other studies reported that delirium onset in the presence of neuropathological processes of dementia contributed for an accelerated cognitive decline superior to that only associated with dementia or delirium (9, 11).

The current study has important limitations. Firstly, it was aimed to collect data of a homogeneous group of patients, available to be assessed with MoCA, and this resulted in a small sample, especially for the delirium groups, reducing the possibility to undergo more complex statistical analyses and to generalize the findings. Specifically, our delirium only group included a very small number of patients, which affects the robustness of the conclusions. We used a cognitive screening test (MoCA) that, despite of being the most widely used screening measure in delirium studies, could have been less sensitive to more severe stages of dementia and delirium superimposed to dementia, when compared to other available tools (e.g., Addenbrooke's Cognitive Examination—III, ACE-III, described recently as a more sensitive tool in describing disease severity levels) (53) not yet tested with delirium patients' samples, but significantly more sensitive than MMSE. Additionally, our sample is constituted by a significant percentage of elderly people with low education which, despite of being representative of the Portuguese reality (16.5% of Portuguese individuals over 65 years old are illiterate and 51% only have basic education–4 years of formal education, according to PORDATA, 2020) may have hampered the cognitive assessment and global data collection, which highlights the need to take in consideration this variable (educational level) when developing assessment protocols for hospitalized individuals with delirium and dementia, when communication is already compromised and low education can amplify these challenges.

Secondly, although we used the IQCODE measure to examine premorbid cognitive status and dementia severity, there was no objective measure of cognitive function of the included participants prior to hospitalization. Also, the lack of a more detailed functional status measure besides the Barthel Index limits a deeper understanding of the sample characteristics. Additionally, in the reassessment at 12-months follow-up, despite we could determine the presence of rehospitalizations or reinfections, we did not examine the presence of changes in prescription, or current use of antipsychotic drugs that could influence the cognitive status pathways of these patients. Future studies would benefit from having a well-characterized cohort of patients with Dementia Severity Rating Scale) (54), although IQCODE has demonstrated moderate sensitivity to dementia severity (55) followed longitudinally with clinical, cognitive, functional, and biological measures, before and after an acute systemic infection.

The results of our study support the need for a stronger monitoring of dementia patients hospitalized with acute infections for the presence of delirium. Increasing the staff awareness for the association between delirium and an increased risk of cognitive deterioration can improve the management of these patients (56). Also, if acute systemic infection is confirmed to be a potential reversible cause of cognitive and functional decline at long term in patients with dementia this represents an initial step in generating novel strategies to prevent and slow the progression or retard the clinical manifestations of neurodegenerative disorders.

## Data Availability Statement

The raw data supporting the conclusions of this article will be made available by the authors, without undue reservation.

## Ethics Statement

The studies involving human participants were reviewed and approved by Ethical Committee of Centro Hospitalar Universitário de Coimbra (Ethics approval Ref. CHUC-065-18). The patients/participants provided their written informed consent to participate in this study.

## Author Contributions

AS, PR, and JC were responsible for data collection, data analyses, and manuscript writing. AC was responsible for manuscript writing review, methods writing, and data analysis. IB and IS contributed to manuscript reviewing and for facilitating data collection. All authors agree to be accountable for the content of the work.

## Funding

This work was financed by the European Regional Development Fund (ERDF), through the Centro 2020 Regional Operational Programme under project CENTRO—01-0145-FEDER-032501 and through the COMPETE 2020—Operational Programme for Competitiveness and Internationalisation and Portuguese national funds via FCT e Fundação para a Ciôncia e a Tecnologia, under project[s] POCI-01-0145—FEDER-032501 and UIDB/04539/2020.

## Conflict of Interest

The authors declare that the research was conducted in the absence of any commercial or financial relationships that could be construed as a potential conflict of interest.

## Publisher's Note

All claims expressed in this article are solely those of the authors and do not necessarily represent those of their affiliated organizations, or those of the publisher, the editors and the reviewers. Any product that may be evaluated in this article, or claim that may be made by its manufacturer, is not guaranteed or endorsed by the publisher.
